# Open Reduction and Internal Fixation of displaced Supracondylar Fractures of Humerus with Crossed K-wires via Medial Approach

**DOI:** 10.5704/MOJ.1407.015

**Published:** 2014-07

**Authors:** S Hussain, S Dhar, A Qayoom

**Affiliations:** Department of Physical Medicine & Rehabilitation,Sheri-Kashmir Institute of Medical Sciences, Soura, India; Department of Physical Medicine & Rehabilitation,Sheri-Kashmir Institute of Medical Sciences, Soura, India; Department of Physical Medicine & Rehabilitation,Sheri-Kashmir Institute of Medical Sciences, Soura, India

## Abstract

This study aimed at evaluating the medial approach for open reduction and internal fixation of Gartland type 3 displaced supracondylar fractures of humerus in children. A prospective, single centre study of on displaced supracondylar humerus fractures in 42 children was carried out at our institute. All fractures were managed with open reduction and internal fixation with crossed K-wires via medial approach. The mean follow-up was 12 months and patients were assessed according to Flynn’s criteria. No patients had post-operative ulnar nerve injury. Cubitus varus was not seen in any patient. Superficial pin tract infection occurred in three patients that subsided with anti-septic dressings and antibiotics. No deep infection occurred. 88.09 % patients showed satisfactory results as per Flynn’s criteria. The medial approach provides an excellent view of the supracondylar area. The approach is convenient due to a lower risk for ulnar nerve injury and better acceptability of the medial incisional scar.

**Key Words:**

Type 3 supracondylar fracture humerus, medial approach, crossed K-wire fixation, medial column comminution

## Introduction

Supracondylar humerus fractures constitute about 17% of all paediatric fractures and are second only to forearm fractures ^1,2^. Most fractures occur between five and eight years of age ^3^. Ninety-eight per cent of the fractures are the extension type ^4^. The widely displaced Gartland type III fractures often carry significant potential for acute complications such as neuro-vascular compromise and compartment syndrome that need immediate attention ^5^ (Table 1). Cosmetic complication in the form of cubitus varus is an important delayed complication.

Closed reduction and percutaneous pinning is the accepted primary treatment modality in the literature ^6,7^. However, closed reduction may fail in situations like delayed presentation and non-availability of imaging facility. In such situations, open reduction and internal fixation with Kirschner wires (K-wires) is a safe procedure to achieve anatomical reduction and maintenance of stable alignment. Various approaches advocated for open reduction are the medial ^8^, lateral ^9^, combined medial and lateral ^10^, posterior ^11^, antero-medial and the anterior transverse. Most surgeons have given up the posterior approach due to the high incidence of elbow stiffness. The antero-medial approach allows visualization of the neurovascular structures and their repair if required. However excessive retraction of wound can injure the ulnar nerve. The lateral approach does not allow complete visualization of the medial column comminution and tilt, resulting in comparatively higher incidence of cubitus varus ^12^. Besides, blind insertion of medial pin can lead to ulnar nerve palsy. Medial approach is routinely used at our institution for open reduction and internal fixation of the widely displaced supracondylar fracture of the humerus. We present our two-year experience with this surgical approach.

## Materials and Methods

**Fig. 1Immediate post-operation Radiograph -showing pin configuration. Note the column comminution.**
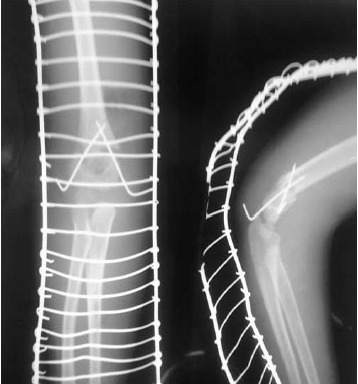


Between August 2008 and August 2010, forty-two (42) patients with Gartland type III supracondylar fractures of the humerus were enrolled in the study (Table 2 ). There were 25 (59.62%) boys and 17 (40.47%) girls. The average age was 8.50 years (range 2–12 years). Majority (76%) of patients reported to department of Accident & Emergency within 24 hours of injury while some reported as late as one week. Forty (95.23%) patients had extension type fracture and two (4.76%) had flexion injury. The mode of trauma was: fall while playing in 18 (42.85%),fall from height in 15 (35.71%) road traffic accidents in 7 (16.66%), blunt trauma in 2 (4.76%) (Table 3). Through a medial approach, all fractures were open reduced and internally fixed with crossed K-wires (Figure 1). Patients with vascular injury were excluded from the study as the approach is dictated by the injured structure. Most patients were operated on within six hours of admission. The average follow-up period was 6 months (Maximum 12 months).

**SURGICAL TECHNIQUE**Surgery was performed under general anaesthesia. With the patient supine, the injured limb was placed on a hand table in abduction and external rotation. Under aseptic precautions and with pneumatic tourniquet, a medial incision was made starting from the medial epicondyle and extending proximally for 3–4 cm. Excess swelling, often associated with these fractures may pose a problem in identifying the bony landmarks. The ulnar nerve, often displaced anteriorly in a flexed elbow, was identified and mobilized to the length of the skin incision. The brachialis and triceps were elevated judiciously from the proximal fragment and the fracture hematoma drained. The entire anterior and medial bone were well visualized. The elbow was flexed and gentle traction applied to disengage and visualize the distal fragment. After achieving as anatomical reduction as possible, a medial K-wire was inserted first to stabilize the fracture. The entry point was the anterior part of medial epicondyle and engaged the posterior cortex of the humerus. The lateral pin was placed from the lateral epicondyle and to engage the opposite cortex . Elbow movements were checked. The wires were cut long to facilitate subsequent removal without anaesthesia. The tourniquet was removed and the wound irrigated with saline. After checking for the capillary refill the subcutaneous tissue and skin were closed. The elbow was immobilized in a splint at 90° flexion in supination. An immediate post-operative check radiograph was obtained (Figure 1). All patients received pre-operative prophylactic antibiotics. The patients were discharged on the third post-op day and asked to return on Day 10 to 12 for suture removal. The cast and K-wires were removed at four weeks and both the parents and children instructed about rehabilitative exercises to be continued at home. Patients with limited elbow range of motion at the end of 6-8 weeks despite this programme were referred to the Physical Medicine and Rehabilitation Clinic. Clinical and radiological assessments were performed at three and six months and at the final visit at 12 months in all patients. The following information were recorded: 1. passive range of elbow motions (flexion/extension, pronation/ supination) 2. loss of range of elbow motion. 3. carrying angles of both sides. 4. Baumann angle and 5. difference in Baumann angle between the radiographs at immediate post-operative and at three months follow-up visit. Results were assessed according to Flynn’s criteria ^6^ (Table 4). In Flynn’s criteria, patients are evaluated according to the functional and cosmetic factors: - loss of flexion or extension clinically, and any deviation of the carrying angle radiologically.

## Results

The appreciation of medial column comminution and tilt enabled an anatomical reduction and alignment in all the cases. There were no ulnar nerve injuries and vascular complications. Thirty-seven (88%) patients regained full range of motion within 6–8 weeks of pin removal. No case of cubitus varus was seen in the present series (Figure 2a and 2b). Post-operative Bauman angle was 16.50 (Range 12 to 23°). The five patients, who had presented late with history of massage. had restricted flexion and extension but no loss of pronation or supination. Three patient developed superficial pin tract infections which resolved with antiseptic dressings Pre-operative median nerve palsy were detected in two patients and both of them resolved in two patients resolved by final follow- up. No myositis ossificans or deep infection was seen. Based on Flynn’s criteria ^6^, 37 (88%) patients had satisfactory outcome while five (12%) had poor results (Table 4).

## Discussion

The management of Gartland type 3 supracondylar fractures is challenging in that no single technique is suitable for all types of fracture. There is no consensus on the timing of surgery, approach for open reduction and configuration of fixation wires ^13^. Closed reduction and per-cutaneous K-wire fixation is the the preferred treatment option when intraoperative imaging facilities is available ^14,15^, but it is associated with 4% to 15% iatrogenic ulnar nerve injury ^16^. Clinically, accurate localization of ulnar nerve by palpation may be misleading, the medially inserted pin always posing a danger to the nerve ^17^. The procedure may be dangerous in presence of swelling in elbow region since surface marking of bony landmarks is difficult. Cubitus varus (Gun stock deformity) is the most common late complication ^9,18,19^ and is usually attributed to medial column comminution and distal fragment tilt. Though medial angulation is difficult to appreciate on immediate postoperative radiographs ^9,18^, measurement of Baumann angle can be reliably used to predict the final carrying angle and consequent success or failure of the closed method and the need for remanipulation ^20^. Closed reduction may fail, particularly in late presentation or after prior manipulation by bonesetters. Open reduction and internal fixation of such widely displaced, irreducible and neglected fractures of the humerus has been accepted option ^14,15^. Although surgical treatment creates risk of infection, the improved outcomes (as per Flynn’s criteria) and decreased risk of neuro-vascular complications outweigh the risk ^21^. It is a safe procedure yielding good results ^14,22^. The risk of myositis ossificans, elbow stiffness and deep infection are seldom seen following open reduction ^14,15,22,23,24,25^.

Weiland reported higher incidence of cubitus varus with the use of lateral approach ^9^. The reason cited is difficulty in judging the medial column pathology ^9^. Surgeons using the posterior approach have also reported a significant incidence of cubitus varus.

The medial approach follows a neutral plane between the brachialis and triceps causing no further damage to virgin tissues around elbow or the neuro-vascular structures anteriorly. Direct visualization of ulnar nerve throughout the length of incision eliminates chances of an iatrogenic injury. Anatomic reduction of the fracture under vision minimizes the chances of malunion. Decompression of the hematoma reduces the risk of compartment syndrome ^26^. Cross K-wire stabilization enables immobilization of the injured elbow at less than 90° flexion, further improving perfusion and venous return.

By about one year, the final results are evident as most of the children regain complete range of motion ^18,25^. Growth disturbance seldom occurs since the fracture involves the metaphysis sparing the epiphysis ^18,19^. Thus late development of an abnormal carrying angle is rarely seen ^22^. Open reduction of the fracture gave us a better understanding of the pathology.

In our study, late presentation was one of the reasons for reduced range of motion in five patients. The average delay in these cases was six days. Two children in our series had repeated manipulations elsewhere and three had undergone massage by traditional doctors. The increase in soft tissue trauma and inappropriate bandaging, cusing oedema and leading to tightening of soft tissues and muscle fibrosis, prevented normal joint motion. Extension loss is mostly due to fibrosis in the torn brachialis.

Sibly et al used the posterior approach and pinned all their 35 cases in retroversion. They still reported a predominant extension loss ^11^. Gruber and Hudson also experienced similar results with the posterior approach ^27^.

Blind pinning may cause iatrogenic ulnar nerve palsy in 2–5% cases ^28,29^. Medial pin insertion is regarded to be a critical step viz a viz ulnar nerve entrapment, a risk associated with this approach ^24^. Iatrogenic nerve palsy requires pin removal, which may compromise the reduction and necessitate repeat reduction and/or exploration. Due to this risk many surgeons place two pins from the lateral side but biomechanical studies have shown this configuration to be inferior to cross pinning ^30^.

(Figure 3)

(Figure 4)

(Figure 5)

**Table I : Gartland Classification of Supracondylar Fractures:**
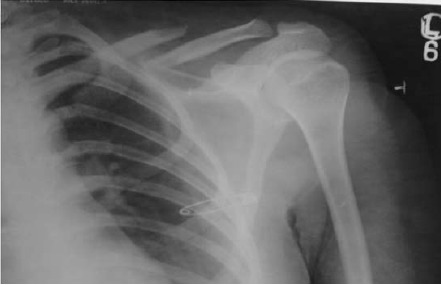


**Table 2 : Patient details and type of Fracture:**
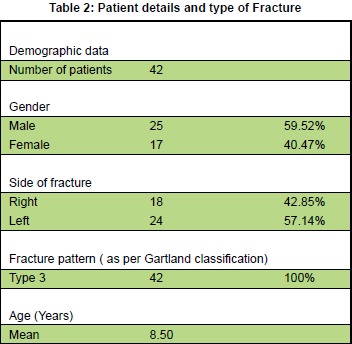


**Table 3 : Mechanism of Trauma:**
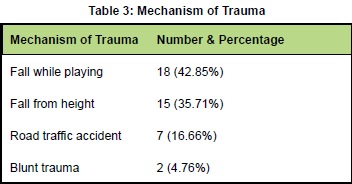


**Table 4 : Results as per Flynn’s criteria:**
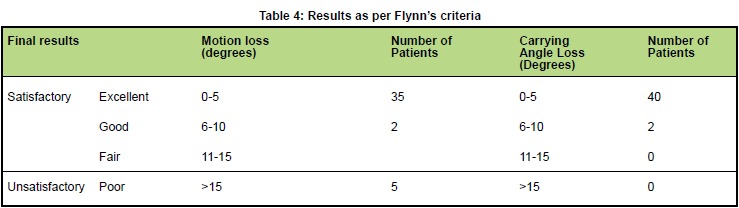


**Fig. 2a: Anterior posterior radiograph at final follow up showing carrying angle:**
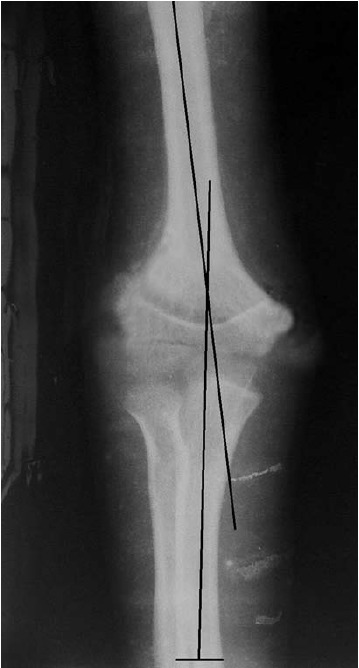


**Fig. 2b: Lateral view radiograph at final follow up showing carrying angle:**
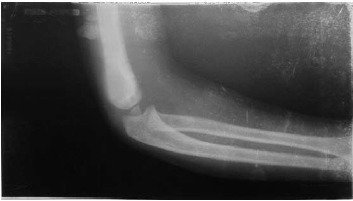


**Fig. 3: Clinical photograph showing carrying angle on operated (R) and normal (L) side.**
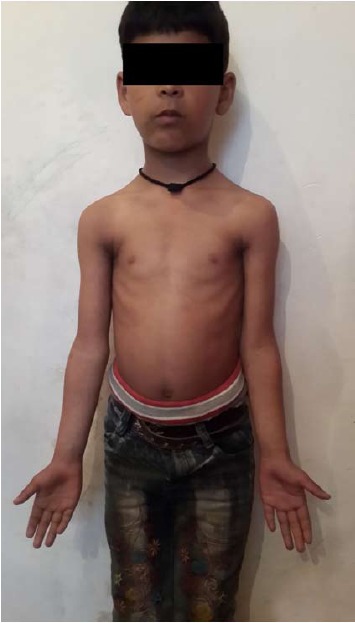


**Fig. 4: Clinical photograph showing full extension on operated (R) elbow.**
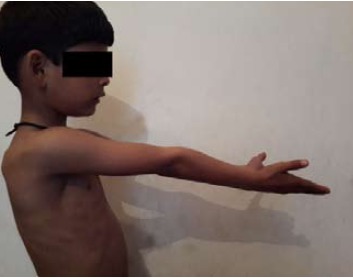


**Fig. 5: Clinical photograph showing full flexion on operated (R) elbow.**
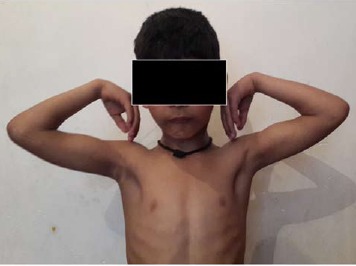


## Conclusion

We conclude that primary operative management of the widely displaced Gartland type 3 supracondylar fracture of the humerus in children using the medial approach is relatively simple and uncomplicated. The approach is safe and easy through the inter-nervous plane. This approach minimizes the chances of an inaccurate reduction and subsequent deformity. Chances of ulnar nerve injury are minimized as the nerve is visualized throughout the length of incision. The functional and cosmetic results are highly satisfactory particularly with regards to the location of surgical scar. The approach is particularly useful when per-operative imaging facilities are not available-
